# How does authentic leadership promote taking charge: The mediating effect of team social capital and the moderating effect of absorptive capacity

**DOI:** 10.3389/fpsyg.2022.1046914

**Published:** 2023-01-05

**Authors:** Guoqing Chen, Jianjian Wang, Zhiyuan Dong, Xiongtao Zhang

**Affiliations:** ^1^School of Economics and Management, University of Science and Technology Beijing, Beijing, China; ^2^School of Information, Beijing Wuzi University, Beijing, China

**Keywords:** authentic leadership, team internal social capital, absorptive capacity, taking charge, proactive behavior, the social learning theory

## Abstract

Authentic leaders are consistent with the expectations of Chinese traditional cultural values for leaders. The purpose of this study is to take team internal social capital (TISC) as the mediating variable and individual absorptive capacity as the moderating variable to explore the relationship between authentic leadership and taking charge. We collected 337 questionnaires from China and used linear regression to explore the influencing mechanisms and boundary conditions. The study results showed that the trickle-down of authentic leadership and the trickle-round of TISC can directly promote employees’ taking charge. Further, individual absorptive capacity positively moderated the relationship between TISC and taking charge. Our study provides a new perspective of leadership support for employees to implement taking charge in the context of high-power distance in China. From the perspective of authentic leadership, it is verified that leadership support is a necessary condition to motivate employees to implement proactive behavior. Moreover, compared with the research on the influence of leadership on proactive behavior, team-related and organization-related factors have not received enough attention. According to the social learning theory, we constructed an integrated framework for the first time in which leadership, team, and individual jointly affect taking charge.

## 1. Introduction

In the digital age, organizations face more uncertainty. Employees take the initiative to change work behavior to constructively complete work, which can effectively promote the functional change of the organization (Grant et al., 2009). It is an important way to enhance organizational response to environmental uncertainty and dynamics. Taking charge refers to that employees take voluntary, proactive, and constructive actions to achieve organizational function change based on change orientation in order to improve the organizational operation (Morrison and Phelps, 1999). Taking charge represents proactive, change or challenge-oriented forms of citizenship (Grant et al., 2011; Zheng et al., 2017). Employees’ proactive change behavior to organizational functions and technical methods can stimulate the creativity, adaptability, and vitality of the organization (Moon et al., 2008; Parker and Collins, 2010). At the same time, taking charge can bring individuals higher performance, job satisfaction, and organizational commitment (Kim et al., 2015; Kim and Liu, 2017).

Leadership behavior is an important situational variable to cultivate employees’ proactive work behavior (Belschak and Den Hartog, 2010). In the Chinese organizational situation, there is a high-power distance between leaders and members. Employees tend to comply with role norms (Farh et al., 2007) and are less willing to implement change and challenging behavior that may offend the authority of leaders (Grant et al., 2011). Which kind of leaders are more likely to support change and challenging behavior becomes a question worth exploring. Authentic leaders refer to leaders whose external behaviors are consistent with their internal values (Gardner et al., 2005). Authentic leaders have good self-cultivation and exemplary roles, which is consistent with the expectations of Chinese traditional cultural values for leaders and has an important influence on the behavior of employees in the organization (Avolio et al., 2004; Hannah et al., 2011; Karadakal et al., 2015). Authentic leadership in the workplace occurs when leaders express their true selves (Leroy et al., 2015). However, the relationship between authentic leadership and taking charge remains to be verified, and the influencing mechanism between them is still unclear.

According to the social learning theory, the behavioral norms of authentic leaders can cause employees to imitate and learn, and their authenticity and openness can promote the exchange of information and resources among team members. Team internal social capital (TISC) represents the team’s ability to acquire information and resources. Authentic leadership may have a positive relationship with TISC. The formation of TISC provides an important condition for individual to propose new technologies, new methods, and problems when they take the initiative to change. In addition, the social learning theory proposes that the observer’s imitative learning is not only affected by the role model, but also by the observer’s willingness and ability to learn (Bandura, 1977). Individuals with strong learning and absorptive capacity can gain more useful clues about resources and technologies from the interaction norms of the group. In other words, the observer’s learning and absorptive capacity may play an important moderating effect between TISC and taking charge.

We have carried out the following studies and made contributions. First, we proposed and verified the positive correlation between authentic leadership and taking charge, and taken TISC as a mediating variable to analyze the influencing mechanism among them. This study provides a new perspective of leadership support for employees to implement taking charge in the situation of high-power distance in China. From the perspective of authentic leadership, it is verified that leadership support is a necessary condition to motivate employees to implement proactive behavior (Crant, 2000). In addition to leader-related factors, Cai et al. (2019) called on researchers to consider more the influence of team or organizational situational factors on proactive behavior. Our study on the relationship between TISC and taking charge is a positive response to the above call. Second, according to the social learning theory, we proposed that absorptive capacity plays a moderating role between TISC and taking charge. For the first time, we have built an integrated framework in which leadership, team, and individual jointly affect taking charge, and verified that those individuals with strong learning and absorptive capacity can obtain more resources and technologies from the interaction norms of the group. It provides key conditions for employees to take the initiative to change.

## 2. Theory and hypothesis

### 2.1. Social learning theory

Bandura’s social learning theory proposes that individuals acquire new things by observing, imitating, and learning the behaviors of others (Bandura, 1977). The learning effectiveness of the observer is related to the demonstrator, observer, and learning situation. For the demonstrators, observers are more likely to learn from those who have great power, high status, and strong ability. For the observers, the observer’s ability, motivation, confidence, and other factors will affect the learning process and effectiveness. In terms of the learning situation, the similarity between the demonstrator and observer will affect the effectiveness of learning. According to the social learning theory, employees learn from leaders, which is seen as trickle-down. Employees learn from team members, which is considered as trickle-round.

### 2.2. Authentic leadership and taking charge

Authentic leaders have the qualities of authenticity, integrity, and fairness, which provide a model for employees to learn and imitate their behaviors. Authentic leadership is regarded as the “root construct” of other positive leadership forms (Avolio and Gardner, 2005). Authentic leaders have a strong sense of self-regulation, show a more open mind to uncertain things and various changes, and will not easily abandon the idea because of its risk (Walumbwa et al., 2008). The inclusiveness and openness of authentic leaders are key characteristics that inspire team members to communicate with each other and share new knowledge and ideas. Neider and Schriesheim (2011) divided authentic leadership into four dimensions: self-awareness, relational transparency, internalized moral perspective, and balanced processing.

Self-awareness is a positive self-concept. Leaders have a clear understanding of their strengths and weaknesses (Zheng et al., 2022) and have an accurate assessment of themselves. Authentic leaders focus on cultivating subordinates’ strengths and broadening their thinking (Avolio and Gardner, 2005; Gardner et al., 2005). Authentic leaders serve as a model for employees to learn and imitate their behavior styles. In the process of communication and behavior learning with authentic leaders, employees will enhance their self-evaluation behaviors and strengths use. Previous research has shown that employees with a high level of strengths use will show more proactive behavior at work (Harzer and Ruch, 2014) and can better adapt to change behavior (Dubreuil et al., 2014).

Relational transparency shows that authentic leaders show themselves to employees in a real and honest way, share information and emotions with employees actively, encourage employees to express their true thoughts and opinions, and can establish a good trust relationship with employees (Gill and Caza, 2018). Chiaburu and Baker (2006) found that there is a positive relationship between trust tendency and employees’ taking charge. Authentic leaders provide an atmosphere for employees to freely express their views and ideas (Gardner et al., 2005). Morrison and Phelps (1999) found that the openness of managers is positively correlated with employees’ taking charge. Openness makes employees face lower risks and higher support for implementing change behavior (Cai et al., 2019). In addition, Leroy et al. (2015) proposed that authentic leaders provide task-related honest feedback to followers, which is intended to promote the growth of followers. This means that subordinates can get task-related valuable information and suggestions from authentic leaders, which provides favorable support for the improvement of subordinates’ task procedures and efficiency.

Internalized moral perspective means that leaders have clear moral standards and values. When facing moral dilemmas and social pressure, they adhere to their moral standards and values as the basis of decision-making. The high standard of internalized ethics of authentic leaders has improved employees’ sense of job security (Borgersen et al., 2014; Zheng et al., 2022). The high internalized moral perspective of authentic leaders can encourage employees to express their views on work issues (Novitasari et al., 2020), which is conducive to increasing employees’ initiative to implement risky change behavior.

Balanced processing means that leaders respect facts and balance various opinions to make decisions in a fair and objective manner (Walumbwa et al., 2008). Such leaders can accept the views of challenging their status (Gardner et al., 2005). Ilies et al. (2005) proposed that unbiased processing is an indicator of psychological authenticity, which can promote authentic leaders to seek challenging situations with learning potential. The learning and challenge orientation will cause the followers to imitate and learn, and then improve the followers’ ability on work tasks (Dweck, 2000), which is helpful to change the working methods and solve problems. Authentic leaders serve as examples of integrity and fairness in behavior (Avolio et al., 2004), Banks et al. (2016) proposed that the information balance processing of authentic leaders is conducive to the followers to obtain the resources, guidance, and help they need to perform their duties. These provide important conditions for followers to take the initiative to change work and effectively solve problems.

*Hypothesis 1*: Authentic leadership is positively correlated with taking charge.

### 2.3. Authentic leadership and team internal social capital

Leaders support is an important condition for the team to acquire resources. TISC is the team’s internal ability to obtain resources and decision-making information, including the structural dimension, cognitive dimension, and relational dimension (Nahapiet and Ghoshal, 1998). In the above analysis, we have learned that authentic leaders can build good relationships with employees. Previous research has suggested that a good relationship can promote mutual trust, thus increasing access to knowledge, information, and other resources (Park and Luo, 2001; Yli-Renko et al., 2001).

The structural dimension mainly refers to the technical exchange and opinion exchange among individuals within the team, the discussion and solution of problems in the team in a constructive way, and the seeking of resources support from the team leaders. Authentic leaders are considered more trustworthy by team members (Gill and Caza, 2018), and team members are more willing to share information openly and truthfully express their thoughts (Gao et al., 2011). Under the guidance of authentic leaders, team members respond positively to organizational improvement (Burris, 2012). Zheng et al. (2022) found that authentic leadership promotes team members to express their opinions on organizational issues. The good trust relationship between the authentic leaders and the team members provides the conditions for technical exchange and the exchange of ideas within the team.

The cognitive dimension mainly refers to that team members share a common vision and goal, and have a clear understanding of the terminology, tools, and methods involved in the team. The good qualities and behaviors of authentic leaders become an example for employees to learn and imitate (Avolio and Gardner, 2005). Team members’ learning and imitation of authentic leaders’ behavior patterns (Zheng et al., 2022) helps team members to build common visions and goals consistent with authentic leaders. Ilies et al. (2005) proposed that authentic leaders transfer their true selves to followers and project their values and visions to followers.

The relationship dimension mainly refers to the mutual trust and understanding among individuals within the team, as well as the new technologies, methods, and ideas proposed by individuals that can be supported. Authentic leaders show themselves to employees truthfully and candidly, take the initiative to share information and emotions with employees, encourage employees to express their true thoughts and opinions, and can establish a good trust relationship with employees (Avolio et al., 2004; Walumbwa et al., 2008). Kernis (2003) found that the openness and authenticity of leaders would lead to a high level of trust between supervisors and subordinates. Ilies et al. (2005) proposed that authentic leaders provide developmental (non-controlling) feedback and support the self-determination of followers. Managers’ support for subordinates’ self-determination promotes subordinates’ trust in the organization (Gagné and Deci, 2005). Authentic leaders’ expression of true feelings will lead to learning among team members and promote trust and support among team members.

*Hypothesis 2*: Authentic leadership is positively correlated with TISC.

### 2.4. The mediating effect of team internal social capital

Social capital can promote the sharing of organizational knowledge and resources and the creation of intellectual capital (Nahapiet and Ghoshal, 1998), providing information and resource conditions for individuals to implement taking charge. Previous research has found that access to information and resources provides opportunities for individuals to implement change-oriented behavior (Fuller et al., 2006; López-Domínguez et al., 2013).

Structured capital and taking charge. Individuals can learn new ideas and technical methods from the technical communication and opinion exchange among team members, which provides useful clues for individuals to change their working ways and develop new technical methods. The discussion of the problems can promote the individual to solve the problems in the team. Tsai and Ghoshal (1998) proposed that internal social capital can promote the exchange and integration of resources within an organization and contribute to the innovation of new product technologies.

Cognitive capital and taking charge. The common vision is a core component of implementing organizational change (Zaccaro and Banks, 2004). The common vision keeps the goals of employees consistent with the goals of the organization, enhances the task confidence of employees, and improves the tendency of employees to take initiative and take risks on work tasks (Choi, 2007). Members are more likely to correct the wrong practices of the team and solve pressing problems. The common vision increases employees’ identification and emotional commitment to the organization, as well as their willingness and enthusiasm to contribute to the organization (Berson et al., 2015). Choi (2007) found that common vision is positively correlated with organizational citizenship behavior for change. Moreover, employees and the team have a common goal and vision, which means that team members are able to act in a collective way. Love and Dustin (2014) found that the collectivism of individual psychology promoted taking charge, and they were more willing to engage in out-of-role behavior that is beneficial to the organization. Inkpen and Tsang (2005) found that a common vision can promote the exchange of knowledge, technologies, and other resources among team members. In the process of resources exchange, employees can obtain more enlightening new technologies and methods, which provide favorable help for proactive work improvement and problem-solving.

Relational capital and taking charge. Trust is the intrinsic basis of relational capital, which reflects the degree of team members are willing to exchange, transfer resources, and cooperate (Granovetter, 1985). Taking charge is risky and challenging behavior. The trust and support of team members can help build an organizational atmosphere of sharing information, challenging the status quo, and daring to change (Wang et al., 2014). Team members will be more active in changing working methods and technologies without worrying too much about the negative effects of change risks (Cropanzano and Mitchell, 2005). Relational capital increases the psychological security of individuals. Ashford et al. (1998) proposed that the support of colleagues in the organization can actively promote the efforts of employees to change. Love and Dustin (2014) confirmed that team member communication and colleague support are positively correlated with taking charge.

*Hypothesis 3*: TISC is positively correlated with taking charge.

*Hypothesis 4*: TISC mediates the relationship between authentic leadership and taking charge.

### 2.5. The moderating effect of absorptive capacity

In the same external knowledge environment, the difference in the ability of individuals or organizations to digest and integrate resources will lead to great differences in the creation of new technologies and methods (Lusch and Nambisan, 2015). According to the social learning theory, the effectiveness of the observer’s learning and imitation is affected by his learning and imitation ability (Bandura, 1977). The observers with strong learning ability can obtain more beneficial technical methods and clues from the TISC, thus promoting the individual’s taking charge. Individual absorptive capacity refers to an individual’s ability to identify, acquire, assimilate/transform and utilize knowledge from the external environment (Cohen and Levinthal, 1989; Todorova and Durisin, 2007; Griffith and Sawyer, 2010).

New technologies, new methods, or knowledge in taking charge are usually complex and unstructured tacit knowledge, which can only be understood and digested after in-depth communication and repeated interaction (Zahra and George, 2002), which puts forward requirements on the knowledge absorption capacity of individuals. TISC promotes technical exchange and opinion exchange among team members and contributes to taking charge. However, individuals with strong absorptive capacity can identify and acquire more beneficial clues in technical communication and experience summary, and understand the deep connotation of the content shared by team members. Khan et al. (2019) proposed that absorptive capacity expands the cognitive ability to assimilate/transform and utilize external knowledge. Absorptive capacity can promote the identification of external opportunities and the generation of new ideas in the process of assimilation/transformation and utilization of external knowledge (Zahra and George, 2002). That is, when an individual has a strong absorptive capacity, he or she can identify, acquire, assimilate/transform and utilize more information or resources related to taking charge in the interaction of TISC. Previous research has indicated that absorptive capacity can promote organizations to acquire more experience and know-how or valuable new knowledge (Kim and Inkpen, 2005; Kafouros et al., 2020). We think this finding holds true in individuals as well.

Team internal social capital provides heterogeneity conditions for the development of new technologies and methods for individuals to implement taking charge, and such heterogeneity requires individuals to have a strong absorptive capacity to understand and integrate acquired information and resources (Lev et al., 2009). By identifying, acquiring, assimilating/transforming, and utilizing existing technologies and abilities, individuals with strong absorptive capacity deepen their existing knowledge base (Wu and Wei, 2013), increase their knowledge reserve, experience and skills, and have a clearer and deeper cognition of professional terms, tools and methods in cognitive capital.

*Hypothesis 5*: Absorptive capacity positively moderates the relationship between TISC and taking charge.

**Figure 1:**
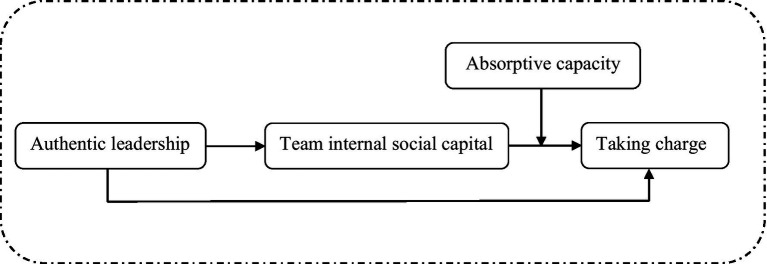
Theoretical model of authentic leadership, TISC, taking charge, and absorptive capacity.

The theoretical model is presented in Figure 1.

## 3. Materials and methods

### 3.1. Samples and data

In May and June 2022, we collected data from Shandong, Beijing, Tianjin, and other provinces and cities in China through both online and offline methods. 475 questionnaires were sent out and 337 valid questionnaires were recovered, with an effective recovery rate of 70.95%. The respondents mainly cover education, training, finance, and other industries. The study was divided into two time points. In the first time point, we collected the data of control variables, authentic leadership, and TISC. Two weeks later, we collected the data of absorptive capacity and taking charge. Pairing was completed through nicknames, mobile phone tail numbers and other ways. The results of the questionnaire showed that males accounted for 39.8% and females accounted for 60.2%. The average age was 30.46 years. 28.2% of them have a junior college degree or below the level, 51.0% have a bachelor’s degree, and 20.8% have a master’s or a doctor’s degree.

### 3.2. Measurement

#### 3.2.1. Authentic leadership

We measured authentic leadership using the 14-item scale developed by Neider and Schriesheim (2011). Examples of the items include “My leader’s inner belief is consistent with his external behavior,” and “my leader openly shares information with others.” All items were rated on a five-point scale, *a* = 0.809.

#### 3.2.2. Taking charge

We measured taking charge using the six-item scale adapted by Li et al. (2015). Examples of the items include “I often try to change the way work is performed to make work more efficient,” “I often try to develop new and more efficient work methods for the company,” and “I often try to introduce new structures, technologies or methods to improve efficiency.” All items were rated on a five-point scale, *a* = 0.899.

#### 3.2.3. Team internal social capital

We measured the structural dimension using the seven-item scale developed by Nahapiet and Ghoshal (1998), the cognitive dimension using the three-item scale developed by Rickards et al. (2001), and the relational dimension using the three-item scale developed by Dakhli and De Clercq (2004). Examples of the items include “Team members can discuss and solve task problems constructively,” “Team members share team vision and know team goals,” and “Team members trust each other and face problems together.” All items were rated on a five-point scale, *a* = 0.923.

#### 3.2.4. Absorptive capacity

We measured absorptive capacity using the nine-item scale developed by Griffith and Sawyer (2010). Examples of the items include “I can identify the most valuable knowledge,” and “It is easy for me to make some job changes to take advantage of the new technical knowledge.” All items were rated on a five-point scale, *a* = 0.790.

#### 3.2.5. Control variables

Demographic characteristics such as gender, education level, and age were used as control variables (Morrison and Phelps, 1999; Kim and Liu, 2017; Zheng et al., 2017). In gender variables, 1 represents female and 2 represents male; Among the variables of education level, 1 means below bachelor’s degree, 2 means bachelor’s degree, and 3 means master’s and doctor’s degrees.

## 4. Results

### 4.1. Confirmatory factor analysis

We performed confirmatory factor analysis using AMOS24.0 to verify the discriminant validity of key variables. The 4-factor model showed $\chi^{2}$ /df = 1.945, GFI = 0.923, AGFI = 0.897, CFI = 0.963, NFI = 0.927, IFI = 0.963, and RMSEA = 0.053, It shows that the key variables involved in this study have good discriminative validity (see Table 1).

**Table 1:** The results of confirmatory factor analysis.

Model	$\chi^{2}$	df	$\;\;\;\chi^{2}$ /df	GFI	AGFI	CFI	NFI	IFI	RMSEA
Four-factor model	250.916	129	1.945	0.923	0.897	0.963	0.927	0.963	0.053
Three-factor model	905.380	132	6.859	0.736	0.659	0.765	0.737	0.766	0.132
Two-factor model	1533.844	134	11.447	0.639	0.539	0.574	0.554	0.577	0.176
Single-factor model	1682.033	135	12.460	0.614	0.511	0.530	0.511	0.532	0.185

Structural equation modeling was used for the analysis. GFI, goodness of fit index; AGFI, adjusted goodness of fit index; CFI, comparative fit index; NFI, normed fit index; IFI, incremental fit index; RMSEA, root-mean-square error of approximation.

Four-factor model: authentic leadership, TISC, absorptive capacity, taking charge.

Three-factor model: authentic leadership, TISC + absorptive capacity, taking charge.

Two-factor model: authentic leadership, TISC + absorptive capacity + taking charge.

Single-factor model: authentic leadership + TISC + absorptive capacity + taking charge.

### 4.2. Hypothesis testing

We used SPSS22.0 software to test the hypothesis. Descriptive statistics and correlation coefficient matrices are shown in Table 2. Regression results showed that authentic leadership was positively associated with TISC in Model 2 (*β* = 0.551, *p* < 0.001), supporting *Hypothesis 2*. Authentic leadership was positively associated with taking charge in Model 4 (*β* = 0.546, *p* < 0.001), supporting *Hypothesis 1*. TISC was positively associated with taking charge in model 5 (*β* = 0.512, *p* < 0.001), supporting *Hypothesis 3* and *Hypothesis 4*. The interaction between TISC and absorptive capacity was significantly related to taking charge in model 6 (*β* = 0.192, *p* < 0.05), supporting *Hypothesis 5* (see Table 3).

**Table 2:** Descriptive statistics and correlation coefficients.

	Mean	SD	1	2	3	4	5	6	7
1. Gender	1.398	0.490	—						
2. Education	1.926	0.697	−0.018	—					
3. Age	30.457	5.052	0.036	−0.072	—				
4. Authentic leadership	3.732	0.541	0.067	0.089	0.003	—			
5. TISC	3.520	0.809	0.125^*^	0.080	0.117^*^	0.381^***^	—		
6. Absorptive capacity	3.767	0.644	0.054	0.083	0.072	−0.071	0.034	—	
7. Taking charge	3.498	0.879	0.143^**^	0.146^**^	0.093	0.356^***^	0.496^***^	0.103	—

*N* = 337. ^***^p < 0.001, ^**^p < 0.01, ^*^p < 0.05.

**Table 3:** Linear regression result.

Variables	TISC		Taking charge			
	Model1	Model2	Model3	Model4	Model5	Model6
Intercept	2.454*** (0.322)	0.543 (0.397)	2.238*** (0.347)	0.343 (0.433)	0.981** (0.332)	−0.252 (0.454)
Gender	0.202* (0.089)	0.160 (0.083)	0.255** (0.096)	0.213* (0.090)	0.151 (0.085)	0.118 (0.083)
Education	0.105 (0.063)	0.066 (0.058)	0.196** (0.067)	0.157* (0.064)	0.142* (0.060)	0.120* (0.058)
Age	0.019* (0.009)	0.019* (0.008)	0.017 (0.009)	0.017 (0.009)	0.008 (0.008)	0.007 (0.008)
Authentic leadership		0.551*** (0.075)		0.546*** (0.082)		0.339*** (0.081)
TISC					0.512*** (0.052)	0.418*** (0.055)
Absorptive Capacity						0.109 (0.063)
TISC* Absorptive Capacity						0.192* (0.082)
*R* ^2^	0.036	0.171	0.052	0.164	0.266	0.316
Adjusted *R*^2^	0.028	0.161	0.044	0.154	0.257	0.301
*F*	4.196***	17.069***	6.109***	16.256***	30.127***	21.674***

*N* = 337. ***p < 0.001, **p < 0.01, *p < 0.05; Values in parentheses are standard errors.

In this study, the SPSS-PROCESS plug-in developed by Hayes was used to test the moderated mediation effect of this model (Hayes, 2013). The results showed that 95% CI did not include 0 when the mean ± 1 SD of the absorptive capacity. The moderated mediating index value was 0.106, 95% CI was [0.033, 0.189], and the interval did not contain 0, indicating that the model had a significant moderated mediation effect (see Table 4).

**Table 4:** Test of moderated mediating effects.

Effect types	Absorptive capacity	Effect	BootSE	BootLLCI	BootULCI
Indirect effect	M − 1SD	0.162	0.051	0.065	0.267
	M	0.230	0.043	0.152	0.319
	M + 1SD	0.298	0.048	0.208	0.396
Index		0.106	0.039	0.033	0.189

**Figure 2:**
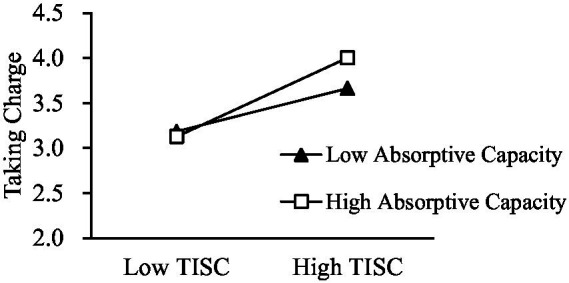
The moderating effect of absorptive capacity on the relationship between TISC and taking charge.

Simple slope analysis showed that no matter whether the absorptive capacity was high (*β* = 0.541, *p* < 0.001) or low (*β* = 0.294, *p* < 0.001), TISC was always positively correlated with taking charge, but the slope of high absorptive capacity was steeper than that of low absorptive capacity (see Figure 2).

## 5. Discussion

### 5.1. Theoretical contributions

At present, the research on OCB mainly focuses on maintaining or strengthening the status quo, while our study focuses on challenging or change-oriented proactive behavior. We construct an integrated framework for influencing taking charge from three aspects of leadership, team, and individual, which provides useful clues for the study of proactive behavior. This study has the following theoretical implications.

First, we proposed and verified the relationship between authentic leadership and taking charge. In the research of the relationship between leaders and taking charge, scholars pay more attention to self-sacrificial leadership, empowerment leadership (Li et al., 2015, 2016; Zhang et al., 2020), etc. Authentic leadership is consistent with the expectations of Chinese traditional cultural values for leaders. However, few scholars have explored the relationship between authentic leadership and taking charge. Our study helps to fill this gap and expand the application of taking charge in the leadership field.

Second, in an organization, the interaction between team members is often more frequent than that between leaders and subordinates (Chiaburu and Harrison, 2008). However, compared with the research on the relationship between leadership and proactive behavior, team-related and organization-related factors have not received enough attention (Cai et al., 2019). Cai et al. (2019) called on researchers to consider more the influence of team and organization situational factors other than the leader-related variable on proactive behavior. Our study, which focuses on the effects both authentic leadership and TISC on taking charge, responds positively to these calls.

Third, current research on employee proactive behavior focuses more on the influence of the motivational effect (Parker et al., 2010). Cai et al. (2019) call for attention to the influence of cognitive and competence factors on proactive behavior. According to the social learning theory, the observer’s learning behavior is influenced by his capacity (Bandura, 1977). We proposed and verified the positive moderating effect of individual absorptive capacity between TISC and taking charge, and reveal the influence of non-motivational effects on proactive behavior.

### 5.2. Practical implications

Our study provides some useful practical implications. First, managers who show their true self can build good trust relationship among employees, provides important psychological support and resources for employees to engage in proactive behavior, and thus promotes employees to engage in out-of-role behavior such as taking charge (Zhang et al., 2015). It enhances the organization’s response to the risks brought by environmental uncertainty.

Second, the organization should enhance the TISC by increasing the interaction among team members, jointly building a common vision and goal, and promoting trust and support among employees. So that employees can learn more information and resources beneficial to enhancing their taking charge through interacting with team members.

Third, employees with strong absorptive capacity can obtain more beneficial resources in team interaction. It means that the organization can improve the ability of employees to identify, absorb and utilize knowledge, which is conducive to the innovation of new technology methods and problem-solving.

### 5.3. Limitations and future research directions

First, although we collected data at multiple time points, it still belongs to cross-sectional data. Longitudinal study design has more advantages in inferring the causal relationship between variables. In future research, cross-period longitudinal data can be used to further make up for this limitation. At the same time, all our variables are from employee self-assessment, which may cause the problem of common method biases. Future research can collect data through multiple assessment methods to reduce the influence of common method biases on research conclusions. Second, based on the social learning theory, our study explored the relationship between leadership, team, individual and taking charge. Scholars can further enrich this framework through other theoretical perspectives and variables. For example, shared leaders advocate that subordinates share leadership responsibilities with leaders, and subordinate members are more willing to take responsibility and take initiative. There may also be a close relationship between shared leadership, TISC and taking charge. Future research can carry out research on this type of leadership. Third, the data of this study are from education, training, and other industries in China. Cross-industry and cross-cultural studies can be carried out in subsequent studies to further enhance the reliability of the conclusions.

## 6. Conclusion

In contrast to promotive and affiliative types of organizational citizenship behavior (OCB), the proactive type of OCB focuses on the future, consciously and actively taking transformative actions to change the work environment (Parker and Collins, 2010). Taking charge is change-oriented behavior and an important way of individual proactive behavior (Choi, 2007). Our study leads to the following conclusions. First, trickle-down of authentic leadership and trickle-round of TISC can directly promote employees’ taking charge. Second, authentic leadership can also promote employees’ taking charge through the indirect transmission effect of TISC. Third, individual absorptive capacity positively moderates the relationship between TISC and taking charge.

## Data availability statement

The original contributions presented in the study are included in the article/supplementary material, further inquiries can be directed to the corresponding author.

## Ethics statement

The studies involving human participants were reviewed and approved by University of Science and Technology Beijing. The patients/participants provided their written informed consent to participate in this study.

## Author contributions

All authors listed have made a substantial, direct, and intellectual contribution to the work and approved it for publication.

## Funding

This study was supported by the General Social Science Project of Beijing Municipal Education Commission (Grant No. SM202210037008) and the School Level Youth Fund Project of Beijing Wuzi University (Grant No. 2022XJQN35).

## Conflict of interest

The authors declare that the research was conducted in the absence of any commercial or financial relationships that could be construed as a potential conflict of interest.

## Publisher’s note

All claims expressed in this article are solely those of the authors and do not necessarily represent those of their affiliated organizations, or those of the publisher, the editors and the reviewers. Any product that may be evaluated in this article, or claim that may be made by its manufacturer, is not guaranteed or endorsed by the publisher.

## References

[ref1] Ashford S. J. Rothbard N. P. Piderit S. K. Dutton J. E. (1998). Out on a limb: the role of context and impression management in selling gender-equity issues. Admin. Sci. Quart. 43:23. doi: 10.2307/2393590

[ref2] Avolio B. J. Gardner W. L. (2005). Authentic leadership development: getting to the root of positive forms of leadership. Leadership Quart. 16, 315–338. doi: 10.1016/j.leaqua.2005.03.001

[ref3] Avolio B. J. Gardner W. L. Walumbwa F. O. Luthans F. May D. R. (2004). Unlocking the mask: a look at the process by which authentic leaders impact follower attitudes and behaviors. Leadership Quart. 15, 801–823. doi: 10.1016/j.leaqua.2004.09.003

[ref4] Bandura A. (1977). Social Learning Theory. Englewood Cliffs, NJ. Prentice-Hall.

[ref5] Banks G. C. McCauley K. D. Gardner W. L. Guler C. E. (2016). A meta-analytic review of authentic and transformational leadership: a test for redundancy. Leadership Quart. 27, 634–652. doi: 10.1016/j.leaqua.2016.02.006

[ref6] Belschak F. D. Den Hartog D. N. (2010). Pro-self, prosocial, and pro-organizational foci of proactive behaviour: differential antecedents and consequences. J. Occup. Organ. Psych. 83, 475–498. doi: 10.1348/096317909x439208

[ref7] Berson Y. Waldman D. A. Pearce C. L. (2015). Enhancing our understanding of vision in organizations. Organ. Psychol. Rev. 6, 171–191. doi: 10.1177/2041386615583736

[ref8] Borgersen H. C. Hystad S. W. Larsson G. Eid J. (2014). Authentic leadership and safety climate among seafarers. J. Leadersh. Org. Stud. 21, 394–402. doi: 10.1177/1548051813499612

[ref9] Burris E. R. (2012). The risks and rewards of speaking up: managerial responses to employee voice. Acad. Manag. J. 55, 851–875. doi: 10.5465/amj.2010.0562

[ref10] Cai Z. Parker S. K. Chen Z. Lam W. (2019). How does the social context fuel the proactive fire? A multilevel review and theoretical synthesis. J. Organ. Behav. 40, 209–230. doi: 10.1002/job.2347

[ref11] Chiaburu D. S. Baker V. L. (2006). Extra-role behaviors challenging the status-quo. J. Manage. Psychol. 21, 620–637. doi: 10.1108/02683940610690178

[ref12] Chiaburu D. S. Harrison D. A. (2008). Do peers make the place? Conceptual synthesis and meta-analysis of coworker effects on perceptions, attitudes, OCBs, and performance. J. Appl. Psychol. 93, 1082–1103. doi: 10.1037/0021-9010.93.5.1082, PMID: 18808227

[ref13] Choi J. N. (2007). Change-oriented organizational citizenship behavior: effects of work environment characteristics and intervening psychological processes. J. Organ. Behav. 28, 467–484. doi: 10.1002/job.433

[ref14] Cohen W. M. Levinthal D. A. (1989). Innovation and learning: the two faces of R & D. Econ. J. 99:569. doi: 10.2307/2233763

[ref15] Crant J. (2000). Proactive behavior in organizations. J. Manage. 26, 435–462. doi: 10.1016/s0149-2063(00)00044-1

[ref16] Cropanzano R. Mitchell M. S. (2005). Social exchange theory: an interdisciplinary review. J. Manage. 31, 874–900. doi: 10.1177/0149206305279602

[ref17] Dakhli M. De Clercq D. (2004). Human capital, social capital, and innovation: a multi-country study. Entrep. Region. Dev. 16, 107–128. doi: 10.1080/08985620410001677835

[ref18] Dubreuil P. Forest J. Courcy F. (2014). From strengths use to work performance: the role of harmonious passion, subjective vitality, and concentration. J. Posit. Psychol. 9, 335–349. doi: 10.1080/17439760.2014.898318

[ref19] Dweck C. S. (2000). Self-theories: their role in motivation, personality and development. Nebr. Sym. Motiv. 38, 199–235. doi: 10.1017/S0021963099226418 2130257

[ref20] Farh J.-L. Hackett R. D. Liang J. (2007). Individual-level cultural values as moderators of perceived organizational support–employee outcome relationships in China: comparing the effects of power distance and traditionality. Acad. Manag. J. 50, 715–729. doi: 10.5465/amj.2007.25530866

[ref21] Fuller J. B. Marler L. E. Hester K. (2006). Promoting felt responsibility for constructive change and proactive behavior: exploring aspects of an elaborated model of work design. J. Organ. Behav. 27, 1089–1120. doi: 10.1002/job.408

[ref22] Gagné M. Deci E. L. (2005). Self-determination theory and work motivation. J. Organ. Behav. 26, 331–362. doi: 10.1002/job.322

[ref23] Gao L. Janssen O. Shi K. (2011). Leader trust and employee voice: the moderating role of empowering leader behaviors. Leadership Quart. 22, 787–798. doi: 10.1016/j.leaqua.2011.05.015

[ref24] Gardner W. L. Avolio B. J. Luthans F. May D. R. Walumbwa F. (2005). Can you see the real me? A self-based model of authentic leader and follower development. Leadership Quart. 16, 343–372. doi: 10.1016/j.leaqua.2005.03.003

[ref25] Gill C. Caza A. (2018). An investigation of authentic leadership’s individual and group influences on follower responses. J. Manage. 44, 530–554. doi: 10.1177/0149206314566461

[ref26] Granovetter M. (1985). Economic action and social structure: the problem of embeddedness. Am. J. Sociol. 91, 481–510. doi: 10.1086/228311

[ref27] Grant A. M. Gino F. Hofmann D. A. (2011). Reversing the extraverted leadership advantage: the role of employee proactivity. Acad. Manag. J. 54, 528–550. doi: 10.5465/amj.2011.61968043

[ref28] Grant A. M. Parker S. Collins C. (2009). Getting credit for proactive behavior: supervisor reactions depend on what you value and how you feel. Pers. Psychol. 62, 31–55. doi: 10.1111/j.1744-6570.2008.01128.x

[ref29] Griffith T. L. Sawyer J. E. (2010). Multilevel knowledge and team performance. J. Organ. Behav. 31, 1003–1031. doi: 10.1002/job.660

[ref30] Hannah S. T. Avolio B. J. Walumbwa F. O. (2011). Relationships between authentic leadership, moral courage, and ethical and pro-social behaviors. Bus. Ethics Q. 21, 555–578. doi: 10.5840/beq201121436

[ref31] Harzer C. Ruch W. (2014). The role of character strengths for task performance, job dedication, interpersonal facilitation, and organizational support. Hum. Perform. 27, 183–205. doi: 10.1080/08959285.2014.913592

[ref32] Hayes A. F. (2013). Introduction to mediation, moderation, and conditional process analysis. J. Educ. Meas. 51, 335–337. doi: 10.1111/jedm.12050

[ref33] Ilies R. Morgeson F. P. Nahrgang J. D. (2005). Authentic leadership and eudaemonic well-being: understanding leader–follower outcomes. Leadership Quart. 16, 373–394. doi: 10.1016/j.leaqua.2005.03.002

[ref34] Inkpen A. C. Tsang E. W. K. (2005). Social capital, networks, and knowledge transfer. Acad. Manag. Rev. 30, 146–165. doi: 10.5465/amr.2005.15281445

[ref35] Kafouros M. Love J. H. Ganotakis P. Konara P. (2020). Experience in R&D collaborations, innovative performance and the moderating effect of different dimensions of absorptive capacity. Technol. Forecast. Soc. 150, 119757–119797. doi: 10.1016/j.techfore.2019.119757

[ref36] Karadakal N. V. Goud N. Thomas P. (2015). Impact of leadership role perspective on conflict resolution styles – a study on small and medium sized entrepreneurs of Karnataka state in India. J. Glob. Entrep. Res. 5, 1–20. doi: 10.1186/s40497-015-0019-6

[ref37] Kernis M. H. (2003). Toward a conceptualization of optimal self-esteem. Psychol. Inq. 14, 1–26. doi: 10.1207/s15327965pli1401_01

[ref38] Khan Z. Lew Y. K. Marinova S. (2019). Exploitative and exploratory innovations in emerging economies: the role of realized absorptive capacity and learning intent. Int. Bus. Rev. 28, 499–512. doi: 10.1016/j.ibusrev.2018.11.007

[ref39] Kim C.-S. Inkpen A. C. (2005). Cross-border R&D alliances, absorptive capacity and technology learning. J. Int. Manag. 11, 313–329. doi: 10.1016/j.intman.2005.06.002

[ref40] Kim T.-Y. Liu Z. (2017). Taking charge and employee outcomes: the moderating effect of emotional competence. Int. J. Hum. Resour. Man. 28, 775–793. doi: 10.1080/09585192.2015.1109537

[ref41] Kim T.-Y. Liu Z. Diefendorff J. M. (2015). Leader-member exchange and job performance: the effects of taking charge and organizational tenure. J. Organ. Behav. 36, 216–231. doi: 10.1002/job.1971

[ref42] Leroy H. Anseel F. Gardner W. L. Sels L. (2015). Authentic leadership, authentic followership, basic need satisfaction, and work role performance. J. Manage. 41, 1677–1697. doi: 10.1177/0149206312457822

[ref43] Lev S. Fiegenbaum A. Shoham A. (2009). Managing absorptive capacity stocks to improve performance: empirical evidence from the turbulent environment of Israeli hospitals. Eur. Manag. J. 27, 13–25. doi: 10.1016/j.emj.2008.04.001

[ref44] Li N. Chiaburu D. S. Kirkman B. L. (2016). Cross-level influences of empowering leadership on citizenship behavior. J. Manage. 43, 1076–1102. doi: 10.1177/0149206314546193

[ref45] Li R. Zhang Z.-Y. Tian X.-M. (2015). Can self-sacrificial leadership promote subordinate taking charge? The mediating role of organizational identification and the moderating role of risk aversion. J. Organ. Behav. 37, 758–781. doi: 10.1002/job.2068

[ref46] López-Domínguez M. Enache M. Sallan J. M. Simo P. (2013). Transformational leadership as an antecedent of change-oriented organizational citizenship behavior. J. Bus. Res. 66, 2147–2152. doi: 10.1016/j.jbusres.2013.02.041

[ref47] Love M. S. Dustin S. L. (2014). An investigation of coworker relationships and psychological collectivism on employee propensity to take charge. Int. J. Hum. Resour. Man. 25, 1208–1226. doi: 10.1080/09585192.2013.826712

[ref48] Lusch R. F. Nambisan S. (2015). Service innovation: a service-dominant logic perspective. MIS Quart. 39, 155–175. doi: 10.25300/misq/2015/39.1.07

[ref49] Moon H. Kamdar D. Mayer D. M. Takeuchi R. (2008). Me or we? The role of personality and justice as other-centered antecedents to innovative citizenship behaviors within organizations. J. Appl. Psychol. 93, 84–94. doi: 10.1037/0021-9010.93.1.84 18211137

[ref50] Morrison E. W. Phelps C. C. (1999). Taking charge at work: extrarole efforts to initiate workplace change. Acad. Manag. J. 42, 403–419. doi: 10.5465/257011

[ref51] Nahapiet J. Ghoshal S. (1998). Social capital, intellectual capital, and the organizational advantage. Acad. Manag. Rev. 23, 242–266. doi: 10.2307/259373

[ref52] Neider L. L. Schriesheim C. A. (2011). The authentic leadership inventory (ALI): development and empirical tests. Leadership Quart. 22, 1146–1164. doi: 10.1016/j.leaqua.2011.09.008

[ref53] Novitasari D. Cahya Kumoro D. F. Yuwono T. Asbari M. (2020). Authentic leadership and innovation: what is the role of psychological capital? Int. J. Sci. Manage. Stud. 3, 27–42. doi: 10.51386/25815946/ijsms-v3i5p103

[ref54] Park S. H. Luo Y. (2001). Guanxi and organizational dynamics: organizational networking in Chinese firms. Strategic Manage. J. 22, 455–477. doi: 10.1002/smj.167

[ref55] Parker S. K. Bindl U. K. Strauss K. (2010). Making things happen: a model of proactive motivation. J. Manage. 36, 827–856. doi: 10.1177/0149206310363732

[ref56] Parker S. K. Collins C. G. (2010). Taking stock: integrating and differentiating multiple proactive behaviors. J. Manage. 36, 633–662. doi: 10.1177/0149206308321554

[ref57] Rickards A. L. Kelly E. A. Doyle L. W. Callanan C. (2001). Cognition, academic progress, behavior and self-concept at 14 years of very low birth weight children. J. Dev. Behav. Pediatr. 22, 11–18. doi: 10.1097/00004703-200102000-00002, PMID: 11265918

[ref58] Todorova G. Durisin B. (2007). Absorptive capacity: valuing a reconceptualization. Acad. Manag. Rev. 32, 774–786. doi: 10.5465/amr.2007.25275513

[ref59] Tsai W. Ghoshal S. (1998). Social capital and value creation: the role of intrafirm networks. Acad. Manag. J. 41, 464–476. doi: 10.5465/257085

[ref60] Walumbwa F. O. Avolio B. J. Gardner W. L. Wernsing T. S. Peterson S. J. (2008). Authentic leadership: development and validation of a theory-based measure. J. Manage. 34, 89–126. doi: 10.1177/0149206307308913

[ref61] Wang Q. Weng Q. McElroy J. C. Ashkanasy N. M. Lievens F. (2014). Organizational career growth and subsequent voice behavior: the role of affective commitment and gender. J. Vocat. Behav. 84, 431–441. doi: 10.1016/j.jvb.2014.03.004

[ref62] Wu A. Wei J. (2013). Effects of geographic search on product innovation in industrial cluster firms in China. Manage. Organ. Rev. 9, 465–487. doi: 10.1111/more.12024

[ref63] Yli-Renko H. Autio E. Sapienza H. J. (2001). Social capital, knowledge acquisition, and knowledge exploitation in young technology-based firms. Strategic Manage. J. 22, 587–613. doi: 10.1002/smj.183

[ref64] Zaccaro S. J. Banks D. (2004). Leader visioning and adaptability: bridging the gap between research and practice on developing the ability to manage change. Hum. Resour. Manag. 43, 367–380. doi: 10.1002/hrm.20030

[ref65] Zahra S. A. George G. (2002). Absorptive capacity: a review, reconceptualization, and extension. Acad. Manag. Rev. 27, 185–203. doi: 10.5465/amr.2002.6587995

[ref66] Zhang X. Li N. Brad Harris T. (2015). Putting non-work ties to work: the case of guanxi in supervisor–subordinate relationships. Leadership Quart. 26, 37–54. doi: 10.1016/j.leaqua.2014.04.008

[ref67] Zhang X. Qian J. Wang B. Chen M. (2020). The role of reward omission in empowering leadership and employee outcomes: a moderated mediation model. Hum. Resour. Manag. J. 30, 226–243. doi: 10.1111/1748-8583.12260

[ref68] Zheng X. Diaz I. Zheng X. Tang N. (2017). From deep-level similarity to taking charge. Leadership Org. Dev. J. 38, 89–104. doi: 10.1108/lodj-06-2015-0134

[ref69] Zheng X. Liu X. Liao H. Qin X. Ni D. (2022). How and when top manager authentic leadership influences team voice: a moderated mediation model. J. Bus. Res. 145, 144–155. doi: 10.1016/j.jbusres.2022.02.073

